# Teaching and Licensing

**Published:** 1880-12

**Authors:** 


### EDITORIAL DEPARTMENT.

"If thou hast truth to utter, speak;
And leave the rest to God."

Teaching and Licensing of physicians should be done by different bodies if competency and thoroughness of training are to be expected at the present time. This matter is assuming greater magnitude and importance every year to both the medical profession, and the laity at large, and it may be well for us to pause and consider it a moment or two now that the year, and the first volume of the Journal are both hastening to a close. The multiplication of Medical Schools has become so great within the decade just past, and the competition among some of the new ones at least, has been so sharp, and active, in order to obtain classes, that low rates, and in a few instances, no rates, of any consequence are required, but assurance given, either expressed, or implied, that the coveted *Diploma* would be forth coming on the completion of a certain brief and inexpensive formula. Some of these " professors " are probably as little capable of appreciating the dignity and value of the medical profession, as many of the so-called students, whom the announcements and *invitations* sent forth by their faculty, seduce from the plow, the forge, and other useful occupations, with which they were formerly contented to remain until the syren notes of M. D. and Diploma were bewitchingly sounded in their ears. If these " professors" must be permitted to expend superfluous vapors before such classes as they are able to beguile into their lecture rooms, they should not be allowed to sit in final judgment on the fitness of candidates for licensure or degrees authorizing them to practice medicine. With the older schools this objection does not obtain, as their faculties are mainly composed of eminent men who care more for their own and the reputation of the institution to which they belong than they do for overrunning the profession with incompetent and illy prepared men, merely to put money in their pockets, and exhibit themselves as professors before the public on commencement days and other occasions. Did any necessity exist for recruiting the ranks of practitioners of medicine, beyond what the old and reputable schools are abundantly able to supply with ease, something might be said in extenuation of the wholesale diploma vending so much deplored at the present day. The following extracts, peculiarly appropriate in this connection, are from an editorial in the *North Carolina Medical Journal*, in which State a *Medical Licensing Body* has been wisely created by the Legislature. "In North Carolina there are exceedingly few irregular physicians, except the usual percentage of morbid growths from the regular stock, and up to this time no eclectic or homœopath has applied for licensure. * * * "The proportion of ill prepared men from a few colleges has attracted the attention of the examiners. It is questionable whether or not the names of these colleges should be given, at any rate the number of their status as judged by their graduates will in no small degree, eventually influence the direction that medical students will go to get their education." At the last examination held in Wilmington there were *seven failures* out of forty candidates. * * * "It is not yet apparent to our state medical examiners that the standard of scholarship has advanced any of late years. If any experience has brought itself home with more force than another it is the propriety of *separating the licensing* from *the teaching bodies.* As long as the present method of bestowing diplomas is followed, the number of rejections by the State Board of Examiners must be greater year by year, for we are satisfied that if a high standard had been erected by the Board at any of the sessions in the last three years, the *number* of *rejections* would have been *twice or thrice* as many as reported. We say all this for the information of those Medical Colleges who are *satisfied* with sending out *unqualified* men in such *large numbers* into this and other Southern States, and also *as advice* to young men who anticipate settling in North Carolina." The foregoing frank and manly extracts, taken from the *North Carolina Medical Journal* confirm in every respect the statements so often made by us on this subject, and show the absolute necessity for *State Medical Examining Boards*, in each and every State, to pass upon the qualifications of all who attempt to practice within their limits.

